# Revealing the effects of various immune cells in anorexia nervosa: Evidence from Mendelian randomization

**DOI:** 10.1097/MD.0000000000041817

**Published:** 2025-03-21

**Authors:** Juan Deng, Yunfeng Yu, Yuman Yin, Gang Hu, Xinyu Yang, Cong Chen, Chenlu Guo, Zhengguo Tang

**Affiliations:** a Department of Anesthesiology, The Third Hospital of Changsha, Changsha, Hunan, China; b Department of Endocrinology, The First Hospital of Hunan University of Chinese Medicine, Changsha, Hunan, China; c School of Traditional Chinese Medicine, Hunan University of Chinese Medicine, Changsha, Hunan, China; d School of Basic Medicine, Guizhou University of Traditional, Chinese Medicine, Guiyang, Guizhou, China.

**Keywords:** anorexia nervosa, immune cells, Mendelian randomization, phenotype, single nucleotide polymorphism

## Abstract

The aim of this study was to assess the causal relationship between immune cells and anorexia nervosa (AN) by Mendelian randomization (MR). Data on immune cell phenotypes and AN were obtained from genome-wide association studies. Next, single nucleotide polymorphisms included in the MR analysis were screened according the basic assumptions. Furthermore, inverse variance weighted was used as the main method for MR analysis to evaluate the causal effect of immune cell phenotypes on AN. Finally, MR-Egger intercept, Cochran *Q*, and leave-one-out sensitivity analyses were used to assess horizontal pleiotropy, heterogeneity, and robustness, respectively. The MR analysis showed that NKT %lymphocyte (OR 1.201, 95% CI = 1.021–1.411, *P* = .027), NKT %T cell (OR 1.258, 95%CI 1.043–1.519, *P* = .017), double negative (DN) (CD4-CD8-) NKT %lymphocyte (OR 1.235, 95%CI 1.074–1.420, *P* = .003), DN (CD4-CD8-) NKT %T cell (OR 1.222, 95%CI 1.060–1.410, *P* = .006), CD8dim NKT absolute count (OR 1.225, 95%CI 1.045–1.436, *P* = .012), CD8dim NKT %lymphocyte (OR 1.214, 95%CI 1.043–1.414, *P* = .012), CD8dim NKT %T cell (OR 1.215, 95%CI 1.035–1.425, *P* = .017), CD16-CD56 on NKT (OR 1.193, 95%CI 1.014–1.402, *P* = .033), CD28 + CD45RA + CD8br %T cell (OR 1.020, 95%CI 1.002–1.037, *P* = .025) were associated with increased genetic susceptibility to AN. MR-Egger showed no horizontal pleiotropy (*P* ≥ .05). Cochran *Q* and sensitivity analysis showed that the results were not heterogeneous and were robust. This MR analysis revealed 9 immune cell phenotypes related to increased genetic susceptibility to AN, emphasizing the importance of NKT and CD8 in AN. This finding provides new insights for understanding the pathogenesis of AN and developing immune-targeted drugs.

## 1. Introduction

Anorexia nervosa (AN) is a multi-factorial functional eating disorder^[1]^ that mainly affects adolescent females.^[2]^ A relevant epidemiological study has shown that the lifetime prevalence of AN is 4% in females and only 0.3% in males.^[3]^ The core characteristics of AN include distorted perceptions of weight and body shape, severe fear of diet and weight control, and strict restrictions on food intake.^[4]^ As the disease worsens, patients with AN may experience acute or chronic complications such as hypoglycemia, anemia, cognitive disorder, cardiac arrhythmia, and coma, which can even be life-threatening.^[5]^ The standardized mortality rate of AN has been reported to be 0.59% per year,^[6]^ ranking first among psychiatric disorders.^[7]^ Although antidepressants play a role in the treatment of AN, their effects are still unsatisfactory.^[8]^ The pathogenesis of AN has not been fully elucidated, and it is generally believed to be related to factors such as genetic factors, social psychology, and interpersonal relationships.^[9,10]^ Therefore, further exploration and comprehension on the pathogenesis of AN are necessary to lay the foundation for the development of cutting-edge AN diagnostic and therapeutic strategies.

Previous studies have suggested that immune cells and cytokines may be involved in the onset or progression of AN.^[11]^ A recent study showed that patients with AN bulimic or purging phenotype had a significantly higher percentage of CD4 T cells and ratio of CD4/CD8 than the general population.^[12]^ Another study found that patients with AN had significantly fewer NK cells and significantly increased levels of sCD40L and sICAM-1 compared to healthy controls.^[13]^ Although previous studies have reported on the association between immune cells and AN, they have mainly focused on a few specific immune cells. In addition, these cross-sectional studies are inevitably subject to the potential influence of confounding variables and reverse causation. Therefore, it is necessary to use novel and effective methods to comprehensively assess the effects of various immune cells on AN.

As an epidemiological research method, Mendelian randomization (MR) is widely used to assess causal associations between exposures and outcomes.^[14]^ Since genetic variation follows a random assignment, MR is less susceptible to confounding variables and reverse causality.^[15]^ In recent years, MR has provided unique insights into the etiology of psychiatric disorders such as schizophrenia and anxiety disorders.^[16,17]^ Therefore, in this study, we used 2-sample MR to assess the causal effect of 731 immune cell phenotypes on the genetic susceptibility to AN.

## 2. Materials and methods

### 2.1. Study design

**Figure 1.**
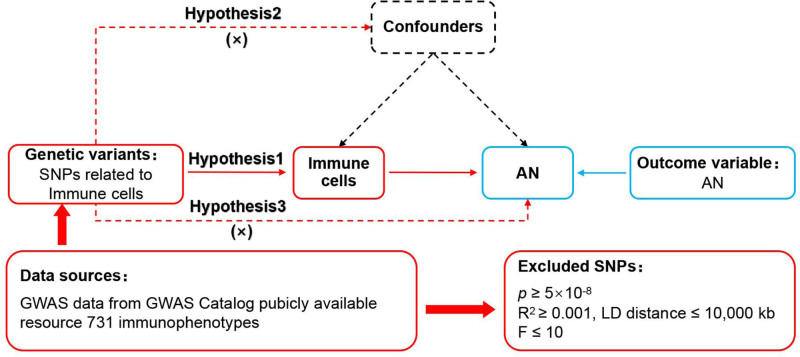
MR design for immune cells on genetic susceptibility to AN. AN = anorexia nervosa, MR = Mendelian randomization.

This MR study was based on 3 basic Mendelian assumptions,^[18]^ as shown in Figure 1. The association assumption required that each single nucleotide polymorphism (SNP) was strongly correlated with exposure. The independence assumption required that each SNP was independent of confounding variables. The exclusivity assumption required that each SNP affected the outcome only through exposure and not through other pathways.

### 2.2. Data sources

Data on immune cell phenotypes numbered from GCST0001391 to GCST0002121 were obtained from the European Bioinformatics Institute (https://www.ebi.ac.uk).^[19]^ The data set has 731 immune cell phenotypes containing a range of immune cells such as Treg cells, mature T cells, B cells, natural killer cells, monocytes, and myeloid cells. Data set for AN with the number ieu-a-1186, which contained genetic information on 14,477 Europeans, was obtained in the Integrative Epidemiology Unit OpenGWAS Database (https://gwas.mrcieu.ac.uk/). As these data were publicly available, the study did not require additional ethical approval.

### 2.3. Selection of genetic instrumental variables

First, *P* < 5 × 10^−8^ was set to search for SNPs closely associated with each phenotype in the data set of immune cells to meet the association assumption. Second, *R*^2^ <0.001 and kb = 10,000 were set to perform a secondary screening of SNPs to exclude interference from linkage disequilibrium. Third, *F* > 10 was set to continue screening SNPs to exclude interference from weakly correlated variables. *F* = [*R*^2^/(1-*R*^2^)]*[(N-*K*-1)/*K*]. *R^2^* referred to the cumulative explained variance of the selected IVs on exposure, N referred to the sample size of the GWAS, and *K* referred to the number of paired samples. Fourth, SNPs containing confounding variables were excluded by the PhenoScanner database to satisfy the independence assumption. Fifth, mismatched SNPs were excluded when adjusting allele orientation. Sixth, significantly biased SNPs (*P* < 1) were excluded using the MR-Pleiotropy Residual Sum and Outlier method to ensure the accuracy of causal inference.

### 2.4. Data analysis

The study followed the STROBE-MR guidelines.^[20]^ TwoSampleMR (0.5.7) of R 4.3.1 software was used for MR analysis. Inverse variance weighted (IVW) was set as the main assessment tool, which enables unbiased causal analysis without pleiotropy. Weighted median and MR-Egger were set as secondary assessment tools, with the former being less sensitive to error values and outliers, and the latter providing effective causal analysis in the presence of pleiotropy. The MR-Egger intercept was used to assess horizontal pleiotropy, with *P* ≥ .05 indicating no significant pleiotropy to satisfy the exclusivity assumption. Cochran *Q* was utilized to assess heterogeneity, with *P* ≥ .05 indicating no significant heterogeneity. Leave-one-out sensitivity analysis was employed to assess the robustness of the MR results, indicating that the results were robust when the combined effect sizes were all on the same side.

## 3. Results

### 3.1. Two-sample MR Analysis

**Figure 2.**
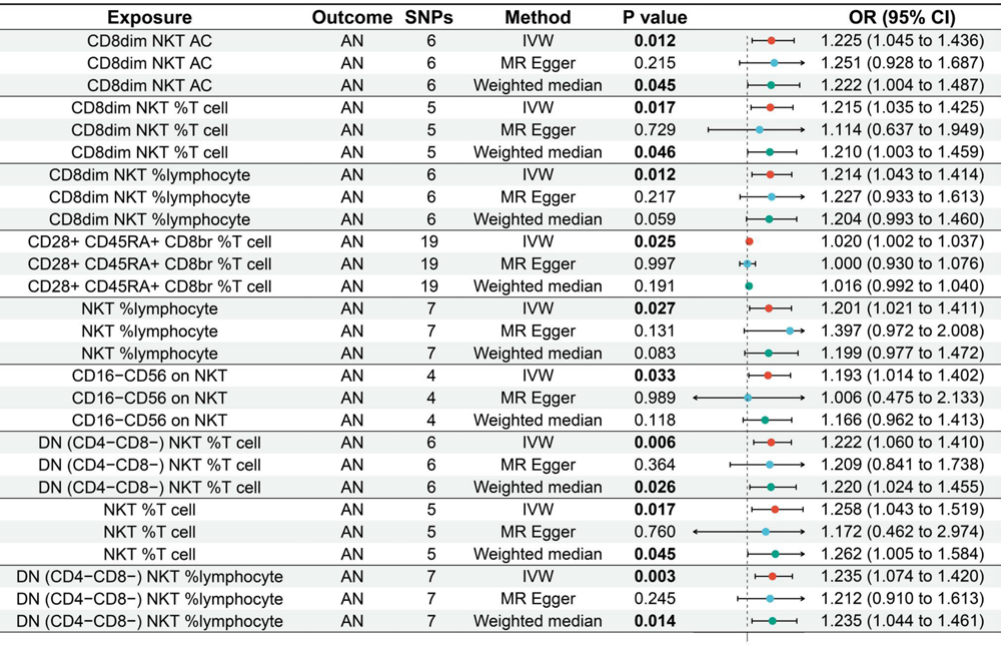
Forest plot of MR analysis for immune cells on genetic susceptibility to AN. AN = anorexia nervosa, MR = Mendelian randomization.

**Figure 3.**
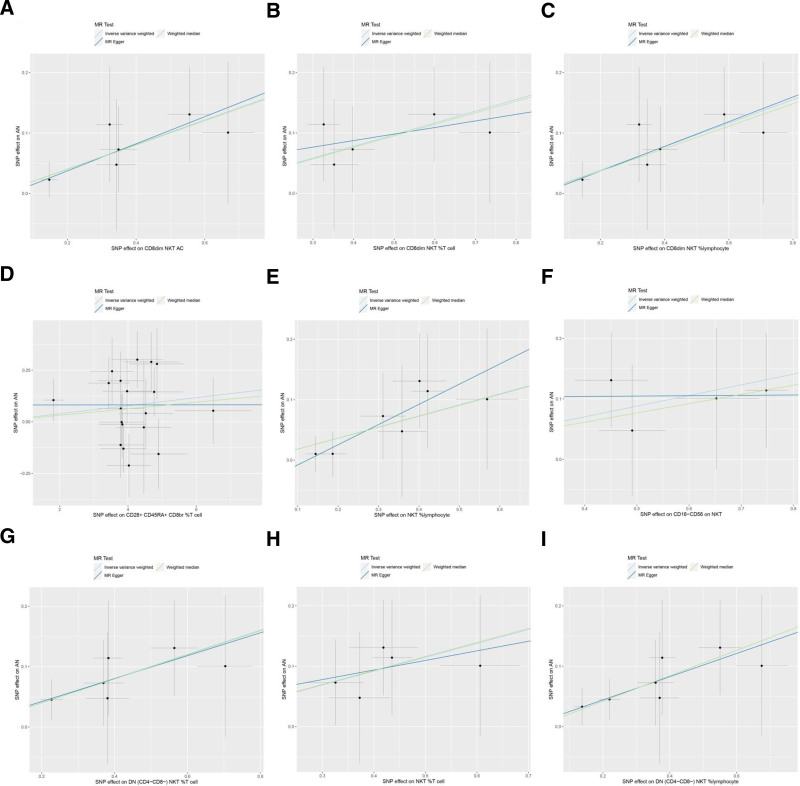
Scatter plot of MR analysis for immune cells on genetic susceptibility to AN. (A) CD8dim NKT AC on AN; (B) CD8dim NKT %T cell on AN; (C) CD8dim NKT %lymphocyte on AN; (D) CD28+CD45RA+CD8br %T cell on AN; (E) NKT %lymphocyte on AN; (F) CD16-CD56 on NKT on AN; (G) DN (CD4-CD8-) NKT %T cell on AN; (H) NKT %T cell on AN; (I) DN (CD4-CD8-) NKT %lymphocyte on AN. AC = absolute count, AN, anorexia nervosa, MR = Mendelian randomization, NKT = natural killer T.

The MR analysis reported that 9 immune cells were associated with the increased genetic susceptibility to AN and the associated SNPs are shown in Tables S1 to S9, Supplemental Digital Content, http://links.lww.com/MD/O540. IVW indicated that CD8dim natural killer T (NKT) absolute count (AC) (odds ratio [OR] 1.225, 95% confidence interval [CI] 1.045–1.436, *P* = .012), CD8dim NKT %T cell (OR = 1.215, 95% CI = 1.035–1.425, *P* = .017), CD8dim NKT %lymphocyte (OR = 1.214, 95% CI = 1.043–1.414, *P* = .012), CD28 + CD45RA + CD8br %T cell (OR = 1.020, 95% CI = 1.002–1.037, *P* = .025), NKT %lymphocyte (OR = 1.201, 95% CI = 1.021–1.411, *P* = .027), CD16-CD56 on NKT (OR = 1.193, 95% CI = 1.014–1.402, *P* = .033), DN (CD4-CD8-) NKT %T cell (OR = 1.222, 95% CI = 1.060–1.410, *P* = .006), NKT %T cell (OR = 1.258, 95% CI = 1.043–1.519, *P* = .017), and DN (CD4-CD8-) NKT %lymphocyte (OR = 1.235, 95% CI = 1.074–1.420, *P* = .003) were associated with increased genetic susceptibility to AN, and the forest and scatter plots are shown in Figures 2 and 3, respectively. The MR-Egger intercepts demonstrated no significant horizontal pleiotropy (*P* ≥ .05), as shown in Table S10, Supplemental Digital Content, http://links.lww.com/MD/O541.

### 3.2. Heterogeneity and sensitivity analysis

**Figure 4.**
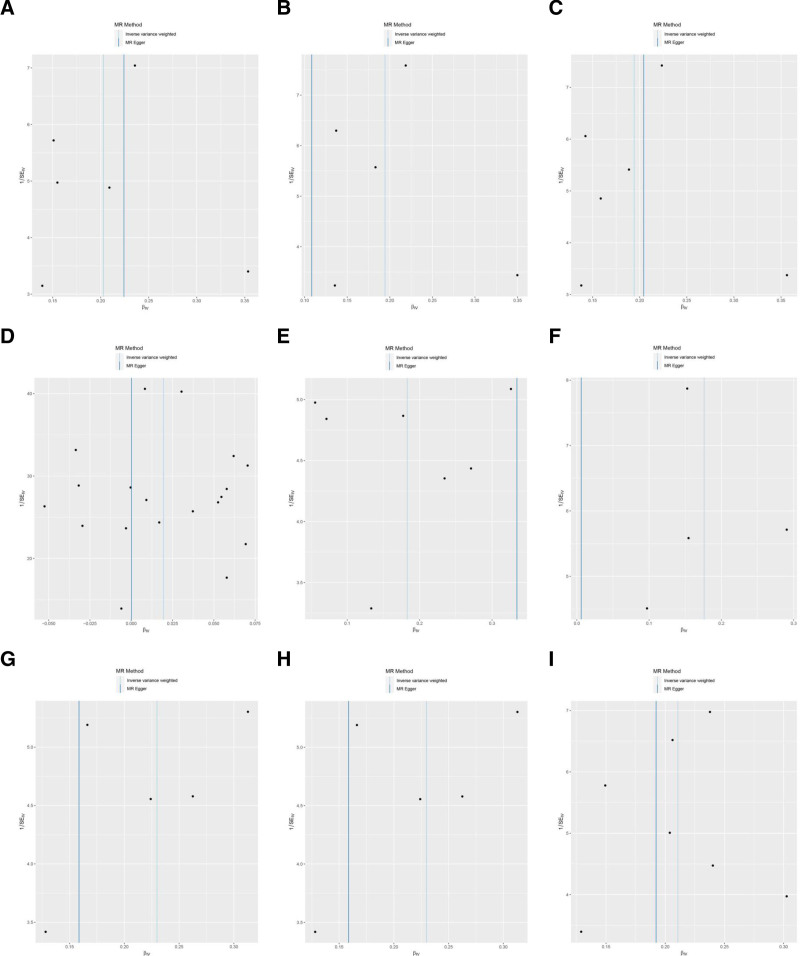
Funnel plot of MR analysis for immune cells on genetic susceptibility to AN. (A) CD8dim NKT AC on AN; (B) CD8dim NKT %T cell on AN; (C) CD8dim NKT %lymphocyte on AN; (D) CD28+CD45RA+CD8br %T cell on AN; (E) NKT %lymphocyte on AN; (F) CD16-CD56 on NKT on AN; (G) DN (CD4-CD8-) NKT %T cell on AN; (H) NKT %T cell on AN; (I) DN (CD4-CD8-) NKT %lymphocyte on AN. AC = absolute count, AN, anorexia nervosa, MR = Mendelian randomization, NKT = natural killer T.

**Figure 5.**
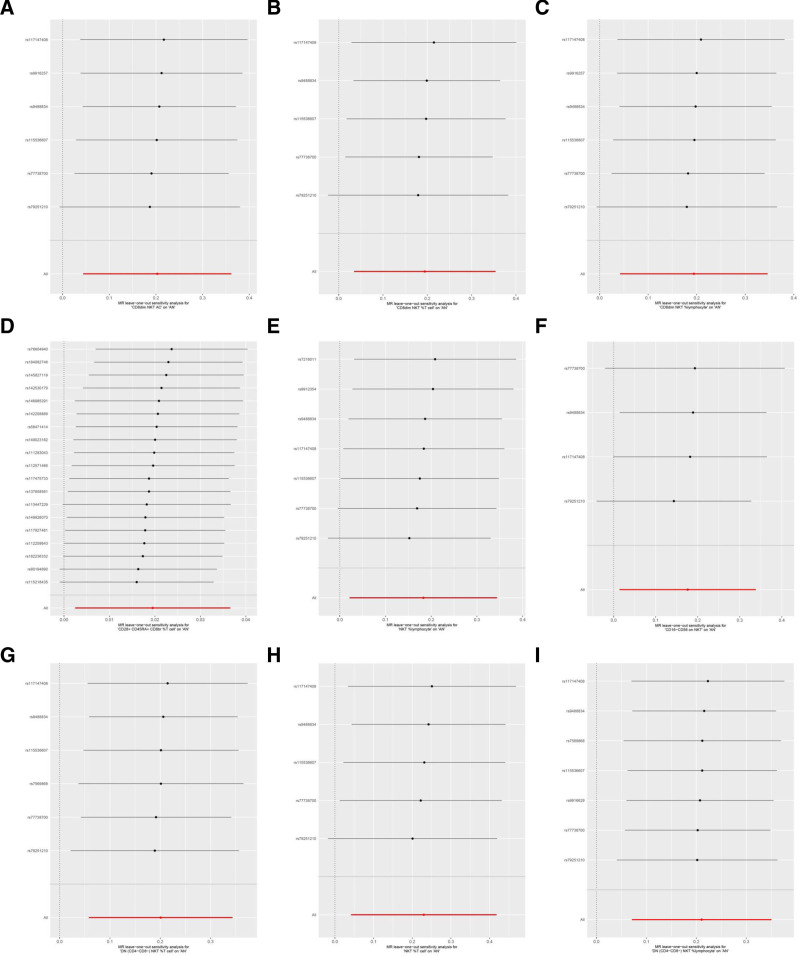
Leave-one-out sensitive analysis for immune cells on genetic susceptibility to AN. (A) CD8dim NKT AC on AN; (B) CD8dim NKT %T cell on AN; (C) CD8dim NKT %lymphocyte on AN; (D) CD28 + CD45RA + CD8br %T cell on AN; (E) NKT %lymphocyte on AN; (F) CD16-CD56 on NKT on AN; (G) DN (CD4-CD8-) NKT %T cell on AN; (H) NKT %T cell on AN; (I) DN (CD4-CD8-) NKT %lymphocyte on AN. AC = absolute count, AN = anorexia nervosa, NKT = natural killer T.

Cochran *Q* showed no heterogeneity (*P* ≥ .05) in the MR results, as shown in Table S11, Supplemental Digital Content, http://links.lww.com/MD/O542 and Figure 4. The leave-one-out sensitivity analysis revealed that the MR analysis results were robust, as shown in Figure 5.

## 4. Discussion

AN is a severe and potentially lethal mental disorder.^[21]^ As the disease progresses, the malnutrition and weight accompanying AN lead to a significant increase in patient mortality.^[22]^ Currently, AN has become a severe public health threat to children and adolescents.^[23]^ Previous studies have suggested that changes in immune cell subsets may be associated with the onset or progression of AN.^[13,24]^ However, limited by the narrow amount of evidence, the specific role of various immune cells in AN is not clear. To our knowledge, this is the first MR analysis to assess the causal effect of 731 immune cell phenotypes on AN using large-scale GWAS data as instrumental variables. Our findings suggest that NKT %lymphocyte, NKT %T cell, DN (CD4-CD8-) NKT %lymphocyte, DN (CD4-CD8-) NKT %T cell, CD8dim NKT AC, CD8dim NKT %lymphocyte, CD8dim NKT %T cell, CD16-CD56 on NKT, and CD28 + CD45RA + CD8br %T cell were associated with increased genetic susceptibility to AN. These results are credible due to the absence of horizontal pleiotropy and heterogeneity.

This MR analysis showed that NKT %lymphocyte, NKT %T cell, DN (CD4-CD8-) NKT %T cell, and DN (CD4-CD8-) NKT %lymphocyte were associated with the increased risk of DN. NKT %lymphocyte and NKT %T cell refer to the percentage of NKT cells in lymphocytes and in T cells, respectively. DN (CD4-CD8-) NKT %lymphocyte and DN (CD4-CD8-) NKT %T cell refer to the percentage of double negative NKT cells in lymphocytes and in T cells, respectively. These immune cell phenotypes indicate that NKT cells play a vital role in the onset or progression of AN. NKT cells are a distinct subset of lymphocytes with innate immune cell and T-cell characteristics, with immune regulation and antitumour effects.^[25,26]^ Double negative NKT is a subset of NKT cells characterized by the expression of CD3 but not CD4 and CD8 co-receptors.^[27]^ Although there are no studies supporting the association of NKT cells and CD3 with AN, some publications have reported their association with other psychiatric disorders such as depression, bipolar disorder, and post-traumatic stress disorder. A clinical study from the Netherlands demonstrated that the number and percentage of NKT cells were significantly higher in elderly depressed patients not using antidepressant medication than those in the general population,^[28]^ suggesting that NKT cells may be a potential risk factor for depression. Subsequently, a cross-sectional study from China included 83 patients with bipolar disorder and 83 patients with major depressive disorder (MDD), and found that CD3 + T-cell count (OR 1.107, 95% CI 1.065 to 1.150) was an independent risk factor for bipolar depression and MDD.^[29]^ Another study from China analyzed selected immune cell phenotypes in 227 patients with unipolar and bipolar depression, and noted that CD3 levels were significantly higher in men in the anhedonia group (2128.69 ± 634.27 vs 1731.86 ± 713.60) compared to the non-anhedonia group.^[30]^ These 2 studies support that CD3 may be a risk factor for mental disorders, suggesting that double negative NKT expressing CD3 may be involved in the onset of AN.

This MR analysis also demonstrated that CD8dim NKT AC, CD8dim NKT %T cell, and CD8dim NKT %lymphocyte were associated with increased genetic susceptibility to AN. CD8dim NKT AC refers to the AC of NKT cells with low expression of CD8. CD8dim NKT %lymphocyte and CD8dim NKT %T cell denote the proportion of NKT cells with low CD8 expression in lymphocytes and in T cells, respectively. These immune cell phenotypes point to CD8dim NKT cells as an essential risk factor for AN, highlighting the crucial role of CD8 and NKT cells. NKT cells are potential risk factors for psychiatric disorders such as depression,^[28]^ as mentioned above. CD8 is 1 of the most common surface markers on T cells, involved in the recognition of antigens by T cells.^[31]^ A clinical study from France demonstrated that patients with AN had significantly higher levels of CD8 + Integrinβ7 + than the healthy population.^[32]^ Another study from Spain, which included 40 patients with AN, showed that patients with AN with a BMI ≤ 17.5 had significantly higher CD8 + levels than the healthy population (28 ± 5 vs 24 ± 4).^[33]^ These studies suggest that CD8 + levels may be positively associated with the risk of AN. Taken together, these findings support CD8dim NKT as a potential risk factor for AN.

CD16- CD56 on NKT is another phenotype of NKT cells that has also been found to be associated with increased genetic susceptibility to AN. CD16- CD56 on NKT refers to NKT cells that do not express CD16 but express CD56. Compared to ordinary NKT cells, CD16- CD56 on NKT cells also express CD56 on their surface. CD56, also known as neural cell adhesion molecule, is mainly expressed on the surface of dendritic cells and NK cells.^[34]^ A clinical study from Italy revealed that patients with type I active bipolar depression had significantly higher counts of CD56 + GMCSF+, CD56 + INFγ+, and CD56 + IL17 + in peripheral blood than those of healthy individuals.^[35]^ Another study from Croatia showed that PTSD patients with POW experience had significantly increased the level of CD56 + P + compared to the healthy individuals at the same baseline (20.6% vs 15.5%).^[36]^ These findings support the association of CD56 with an increased risk of psychiatric disorders, suggesting that CD16-CD56 on NKT may be a potential risk factor for AN.

In addition to the immune cell phenotypes described above, our findings suggest a potential relationship between CD28 + CD45RA + CD8br %T cells and AN. CD28 + CD45RA + CD8br %T cells represent the proportion of CD28-expressing, CD45RA-expressing, and CD8-highly-expressing cells among T cells. CD8+ may be a potential risk factor for AN,^[33]^ as mentioned above. CD28 is a co-stimulatory molecule on the surface of T cells that provides a second signal for T cell activation by binding to the T cell receptor.^[37]^ CD45RA is a subtype of the CD45 molecule that participates in T cell differentiation.^[38]^ Although there is a lack of relevant literature confirming the association of CD28 or CD45RA with AN, available studies support the association of CD28 and CD45RA with other psychiatric disorders. An animal study showed that CUMS depressed mice had higher levels of CD45+ compared to normal mice.^[39]^ Another clinical study in the United States demonstrated that after adjusting for sociodemographics, smoking, and substance use, patients with PTSD had a significantly higher proportion of CCR7-CD45RA + CD27-CD28- to CCR7 + CD45RA + CD27 + CD28 + than the non-PTSD group (β 1.52, 95% CI 0.56–2.48).^[40]^ These findings support the association of CD28 and CD45RA with psychiatric disorders and indirectly suggest that CD28 + CD45RA + CD8br %T cell may be a potential risk factor for AN.

Although this MR analysis enriches the genetic evidence for the association between immune cell phenotype and AN, there are still some limitations: First, since the datasets included in this study were all from individuals of European ancestry, this finding mainly explains the impact of immune cells on the genetic susceptibility of AN in Europeans. However, considering the genetic susceptibility differences across ethnicities, further research is needed on the impact of immune cells on other ethnic groups, such as African and Asian populations. Second, there may be unrecognized pathways or confounding variables between exposure and outcome, which may increase the risk of bias in the results. Third, although this MR revealed 9 immune cell phenotypes associated with increased genetic susceptibility to AN, it could not explain the molecular biological mechanisms by which they induce or aggravate AN.

We expect future research to make the following efforts to address these limitations: First, future studies should extend these findings by exploring diverse populations beyond those of European ancestry. This will help validate the generalizability of our results and identify any population-specific differences in the relationship between immune cell phenotypes and AN risk. Second, longitudinal analyses, such as prospective cohort studies, are essential to validate the impact of these immune cell phenotypes on the risk of AN over time. Such studies can provide insights into the dynamic changes in immune cell profiles during the onset and progression of AN. Finally, further investigation of the biological pathways involved is crucial. Animal experiments and mechanistic studies are needed to elucidate the precise molecular mechanisms by which these immune cell phenotypes induce or exacerbate AN. This will be instrumental in developing precise diagnostic tools and therapeutic strategies targeting the immune system.

## 5. Conclusion

This study demonstrates that NKT %lymphocyte, NKT %T cell, DN (CD4-CD8-) NKT %lymphocyte, DN (CD4-CD8-) NKT %T cell, CD8dim NKT AC, CD8dim NKT %lymphocyte, CD8dim NKT %T cell, CD16-CD56 on NKT, and CD28 + CD45RA + CD8br %T cell were associated with increased genetic susceptibility to AN. It highlights the importance of NKT and CD8 for AN, and provides new insights for understanding the pathogenesis of AN and developing immune-targeted drugs. However, more studies are necessary in the future to validate this result and explore the underlying biological mechanisms.

## Author contributions

**Conceptualization:** Juan Deng, Zhengguo Tang.

**Data curation:** Yunfeng Yu.

**Methodology:** Yunfeng Yu.

**Formal analysis:** Yuman Yin.

**Supervision:** Chenlu Guo, Zhengguo Tang.

**Writing – original draft:** Juan Deng, Yunfeng Yu, Yuman Yin, Gang Hu, Xinyu Yang, Cong Chen.

**Writing – review & editing:** Chenlu Guo, Zhengguo Tang.

## Supplementary Material

SUPPLEMENTARY MATERIAL
